# A Combination of Ex Vivo and In Vivo Strategies for Evaluating How Much New Oral Anticoagulants Exacerbate Experimental Intracerebral Bleeding

**DOI:** 10.1055/s-0043-1770782

**Published:** 2023-07-10

**Authors:** Juliana R. P. Ferreira, Isabela D. Sucupira, Gabriella M. C. Carvalho, Fernando F. Paiva, Pedro M. Pimentel-Coelho, Paulo H. Rosado-de-Castro, Paulo A. S. Mourão, Roberto J. C. Fonseca

**Affiliations:** 1 Laboratório de Tecido Conjuntivo, Hospital Universitário Clementino Fraga Filho and Programa de Glicobiologia, Instituto de Bioquímica Médica Leopoldo de Meis, Centro de Ciências da Saúde, Universidade Federal do Rio de Janeiro, Rio de Janeiro, RJ, Brazil; 2 Laboratório de Coagulação e Trombose, Hospital Universitário Clementino Fraga Filho, Instituto de Ciências Biomédicas, Centro de Ciências da Saúde, Universidade Federal do Rio de Janeiro, Rio de Janeiro, RJ, Brazil; 3 Centro de Imagens e Espectroscopia por Ressonância Magnética (CIERMag). Departamento de Física e Ciência Interdisciplinar. Instituto de Física de São Carlos, Universidade de São Paulo, São Carlos, Brazil; 4 Laboratório Intermediário de Neuropatologia Experimental. Instituto de Biofísica Carlos Chagas Filho, Centro de Ciências da Saúde, Universidade Federal do Rio de Janeiro, Rio de Janeiro, RJ, Brazil; 5 Laboratório Intermediário de Neuropatologia Experimental. Instituto Nacional de Ciência e Tecnologia em Medicina Regenerativa, Rio de Janeiro, Brazil

**Keywords:** intracerebral hemorrhage, apixaban, rivaroxaban, dabigatran, warfarin

## Abstract

**Background** Intracerebral hemorrhage is the most serious complication of anticoagulant therapy but the effects of different types of oral anticoagulants on the expansion of these hemorrhages are still unclear. Clinical studies have revealed controversial results; more robust and long-term clinical evaluations are necessary to define their outcomes. An alternative is to test the effect of these drugs in experimental models of intracerebral bleeding induced in animals.

**Aims** To test new oral anticoagulants (dabigatran etexilate, rivaroxaban, and apixaban) in an experimental model of intracerebral hemorrhage induced by collagenase injection into the brain striatum of rats. Warfarin was used for comparison.

**Methods** Ex vivo anticoagulant assays and an experimental model of venous thrombosis were employed to determine the doses and periods of time required for the anticoagulants to achieve their maximum effects. Subsequently, volumes of brain hematoma were evaluated after administration of the anticoagulants, using these same parameters. Volumes of brain hematoma were evaluated by magnetic resonance imaging, H&E (hematoxylin and eosin) staining, and Evans blue extravasation. Neuromotor function was assessed by the elevated body swing test.

**Results and Conclusions** The new oral anticoagulants did not increase intracranial bleeding compared with control animals, while warfarin markedly favored expansion of the hematomas, as revealed by magnetic resonance imaging and H&E staining. Dabigatran etexilate caused a modest but statistically significant increase in Evans blue extravasation. We did not observe significant differences in elevated body swing tests among the experimental groups. The new oral anticoagulants may provide a better control over a brain hemorrhage than warfarin.

## Graphical abstract

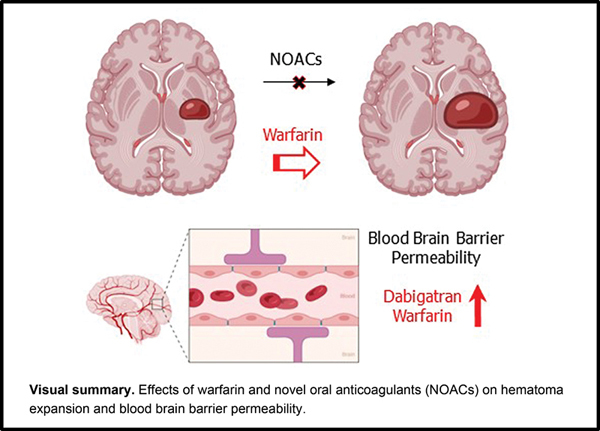

## Introduction

The main treatment for acute venous thromboembolism (VTE) is anticoagulant therapy with fast-acting and parenterally administered drugs such as unfractionated heparin, low-molecular-weight heparin, or the pentasaccharide fondaparinux. They prevent the extension of thrombus and its consequences. 1 In parallel, long-term prophylaxis with oral anticoagulants, such as warfarin or the new oral anticoagulants (NOACs) has been employed. 2 These oral drugs vary in their mechanism of action. Dabigatran etexilate is a direct thrombin inhibitor while apixaban and rivaroxaban are factor Xa inhibitors. Available since the 1940s, warfarin acts as a vitamin K antagonist and is a popular choice for treating VTE. Intracerebral hemorrhage (ICH) is among the most serious and adverse complications caused by these drugs. 3 ICH becomes a relevant focus of study especially because we have little information about its pathophysiology.

Despite its various limitations, warfarin remains the most used oral anticoagulant due to its low cost. However, it carries a high risk of ICH. 4 In patients over 50 years old undergoing warfarin treatment, the risk is up to eight times greater than in untreated patients and the mortality rate can reach 62% within 30 days. 5

The expansion of ICH induced by NOACs remains a topic of intense debate. Although some studies show a low probability of ICH expansion with the NOACs, 6 7 8 9 10 other reports indicate that the risk of ICH associated with the use of these anticoagulants is similar to that of warfarin. 11 12 13 This topic remains in debate even among the different types of NOACs. A retrospective study with 118,891 patients with atrial fibrillation found that treatment with rivaroxaban was associated with a statistically significant increase in cases of ICH compared with dabigatran etexilate. 14 Meta-analysis studies comparing NOACs with warfarin in terms of risk of intracerebral bleeding, hematoma volume, and outcomes showed that bleeding risk depends on the ethnics, type of anticoagulant employed, dose used, and patient-related variables such as age, sex, and comorbidities. 15 16 17

**Fig. 1:**
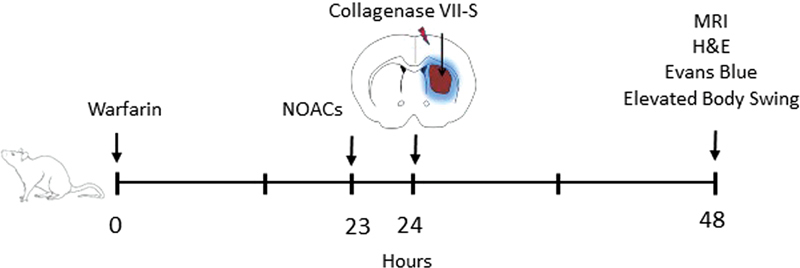
Experimental approach used to assess the effect of NOACs and warfarin on the expansion of ICH. The anticoagulants were orally administered to rats (1 or 1.5 mg/kg of warfarin, 18 mg/kg of apixaban or rivaroxaban, or 9 mg/kg of dabigatran etexilate). After the period required for the anticoagulant to achieve its maximum effect (∼1 hour for the NOACs and 24 hours for warfarin), collagenase (0.4 U) was injected into the left brain striatum. After 24 hours, the brains of the animals were analyzed by MRI, histological examinations, and Evans blue extravasation. Functional neurological assessment was performed using the elevated body swing test. MRI, magnetic resonance imaging; NOACs, new oral anticoagulants.

Experimental models of ICH provide alternatives to identify the mechanisms of injury and to evaluate possible reparative therapies, since there is no specific treatment for patients with this clinical condition, especially those with severe tissue damage. 18 In this study, we compared the effects of NOACs and warfarin on the expansion of ICH using an experimental model of cerebral bleeding induced by collagenase injection into the brain striatum of rats. 19 The enzyme digests collagen present in the basal lamina that surrounds blood vessels, leading to rupture and bleeding into adjacent tissues. This model reproduces experimentally the effects of spontaneous ICH in humans. 20 Equivalent doses and periods of time required for the different anticoagulants to achieve their maximum effects were identified by appropriate ex vivo clotting assays and an in vivo venous thrombosis model. Fig. 1 summarizes our experimental approach. Anticoagulants were administered via oral route followed by collagenase injection into the brain. After 24 hours the sizes of the hematomas were evaluated by ex vivo magnetic resonance imaging (MRI), histological examination, in vivo Evans blue extravasation, and elevated body swing test.

## Material and Methods

### Oral Administration of Anticoagulants in Rats

Warfarin, rivaroxaban, dabigatran etexilate, and apixaban were from Chemphar Co., Ltd, Nanjing, China. Fasted Wistar rats (both sexes, approximately 300 g body weight) were randomly divided into various groups. NOACs or warfarin were dissolved in 500 µL of appropriate solution and administered by gavage. Dabigatran etexilate and warfarin were dissolved in phosphate-buffered saline. This solution was then stirred at 300 rpm for 30 minutes at 40°C. Rivaroxaban and apixaban were dissolved in polyethylene glycol/H _2_ O/glycerol (996:100:60, g/g). Thereafter, the animals were subjected to ex vivo anticoagulant assay and in vivo venous thrombosis test, as described below. We followed the institutional guidelines for animal care and experimentation (approval protocol number 115/19).

### Ex Vivo Anticoagulant Action

Citrated blood samples were collected (nine parts blood: one part 3.8% sodium citrate, v/v) using a cannula inserted into the carotid artery of Wistar rats at different intervals of time after the oral administration of the anticoagulants. The following clotting assays were performed according to the manufacturer's specifications: activated partial thromboplastin time (Biolab-Merieux AS, Rio de Janeiro, Brazil), thrombin time (TT; 5 NIH U/mL human thrombin; Diagnostica Stago, Asnières, France), and prothrombin time (PT) with polybrene free thromboplastin (Diagnostica Stago). The anticoagulant activities were expressed as *T*_1_ / *T*_0_ , which is the ratio between the clotting time in the presence and absence of anticoagulants in the sample.

### Venous Thrombosis

Antithrombotic activity in rats was assessed using rabbit brain thromboplastin as the thrombogenic stimulus. 21 The rats (both sexes, approximately 300 g body weight, seven animals per dose) were anesthetized with an intramuscular injection of 100 mg/kg body weight of ketamine (Cristália, São Paulo, Brazil) and 16 mg/kg body weight of xylazine (Bayer AS, São Paulo, Brazil). Different doses of the anticoagulants were administered orally. At the peak of anticoagulant activity, the inferior vena cava was isolated, and brain thromboplastin (5 mg/kg) from Biolab-Merieux AS, Rio de Janeiro, Brazil, was slowly injected intravenously. After 1 minute, 0.7 cm of the isolated vena cava was clamped using distal and proximal sutures. After 20 minutes of stasis, the thrombus formed inside the occluded segment was carefully removed, washed with phosphate-buffered saline, dried for 24 hours at room temperature, and weighed.

### Bleeding Effect

Wistar rats (both sexes, approximately 250 g body weight) were anesthetized with a combination of xylazine and ketamine, as described earlier. 22 Different doses of the anticoagulants were orally administered, and at the peak of anticoagulant activity, the tail was cut 5 mm from the tip and carefully immersed in 40 mL of distilled water at room temperature. After 60 minutes, the blood loss was determined by measuring the amount of hemoglobin dissolved in water using a spectrophotometric method. The volume of blood lost was deduced from a standard curve based on the absorbance at 540 nm.

### ICH Model

We used the model of collagenase-induced ICH developed by Rosenberg et al, 19 with minor modifications. The animals were anesthetized through an intramuscular injection of xylazine hydrochloride (15 mg/kg) and ketamine hydrochloride (100 mg/kg), and then were placed in a stereotaxic apparatus. The bregma was located, and a hole approximately 1.5 mm in diameter was drilled in the skull to allow the introduction of a 26-gauge needle attached to a 2-mL syringe (Hamilton) into the left striatum (3 mm lateral to midline, 0.2 mm posterior to bregma, 6 mm below the surface of the skull). Two microliters of sterile saline containing 0.4 U of bacterial collagenase VII-S (Sigma-Aldrich) or the same volume of the vehicle (sham group) was then injected over 3 minutes. After infusion, the needle was left in place for 10 minutes, and then withdrawn slowly to prevent backflow. The burr hole was sealed with dental cement, the skin was sutured, and rats were placed in clean cages with free access to food and water. Normothermia was maintained during and after surgery using a heating pad and heating lamps. Twenty-four hours after the induction of ICH, animals were subjected to several evaluations.

### Ex Vivo Magnetic Resonance Imaging and Volumetry

Ex vivo MRI was used to measure the hematoma volume. Rats were deeply anesthetized through an intraperitoneal injection of a mixture of xylazine hydrochloride (15 mg/kg) and ketamine hydrochloride (100 mg/kg), and then perfused through the heart with ice-cold 0.9% saline, followed by 4% paraformaldehyde (PFA) in phosphate buffer, pH 7.4. The heads were kept in 4% PFA at 4°C until image acquisition. MRI was performed in a 2.0 Tesla magnetic resonance system composed of an Oxford Instruments 85310HR Magnet (Oxford Instruments) and Bruker Avance AVIII console (Bruker-Biospin). A locally developed solenoid coil was used as a transmission and reception coil. T1-, T2-, and T2*-weighted 3D sequences were acquired using the same geometric parameters: FOV = 32 × 32 × 32 mm with a matrix of 128 × 128 × 128, resulting in an isotropic spatial resolution of 250 mm. The images were reconstructed using zero filling for a 256 × 256 × 256 matrix. T1-weighted images were acquired with a gradient echo sequence (GRE) with the following parameters: time to echo(TE)/repetition time (TR) = 15/3.5 ms, flip angle = 30°, bandwidth= 15 kHz. T2-weighted images were acquired using a reverse fast imaging with steady-state free precession (PSIF) sequence with the following parameters: TR/TE: 24/7 ms, flip angle = 60°, bandwidth = 15 kHz. Finally, T2*-weighted images were acquired with a GRE sequence of the following parameters: TE/TR = 20/10.5 ms, flip angle = 5°, bandwidth = 15 kHz. The volumes of residual hematoma and of cerebral hemispheres were calculated using the Medical Image Processing, Analysis and Visualization application (MIPAV 8.0.2; National Institutes of Health), performed by an examiner blinded to treatment allocation. Hematoma contour was extracted by manually outlining the regions of hyperintensity and hypointensity, which were distinct from the surrounding tissues in each image. To evaluate the residual hematoma volume, the region of interest was marked around the residual hematoma in all coronal sections where it was visible.

### Evans Blue Quantification Assay

Anticoagulated and control Wistar rats (both sexes, approximately 300 g body weight, eight animals per type or dose of anticoagulant) were subjected to the ICH model described above and, after 24 hours, 1 mL of 4% Evans blue dye was injected into the abdominal vena cava of each animal, previously anesthetized. After 30 minutes, the animals were perfused as described above, decapitated, and the brain was macerated and diluted in 3 mL of formamide and then kept at 37°C for 72 hours. After this period, the amount of Evans blue extravasation from the brain into the supernatant was quantified based on a standard curve in the spectrophotometer at *A*_620 nm_ .

### Histological Analysis

Animals were deeply anesthetized with xylazine/ketamine and then perfused through the heart with 0.9% ice-cold saline, followed by 4% PFA (pH 7.4). Brains were rapidly removed from skulls, post-fixed in 4% PFA for 1 day at 4°C, and cryoprotected overnight in the same solution, but now containing 30% (w/v) sucrose. Frozen brains were sectioned into 20-μm-thick coronal sections using a sliding microtome (Leica Biosystems). Slices were collected in a cryoprotectant solution (0.05 mol/L sodium phosphate buffer, pH 7.4, 30% ethylene glycol, 20% glycerol) and analyzed by standard hematoxylin:eosin (H&E) staining protocol. Slides were scanned with a Panoramic MIDI II scanner (3DHISTECH Ltd., Budapest). The analysis of the images and the quantification of the areas with lesions were performed using the CaseViewer 2.2, 64-bit version program (3DHISTECH).

### Elevated Body Swing Test

The elevated body swing test was performed by an examiner blinded to the treatment allocated to the animal for the measurement of asymmetrical motor behavior, as described by Borlongan and Sanberg. 23 The tests were performed 1 day before and 24 hours after the induction of ICH in treated and nontreated groups. In brief, animals were suspended in the air by the tail, and the direction of body swing (‘‘a >10° bending of the upper body out of the vertical axis to either side’') was recorded in four blocks of eight consecutive trials, with 1 minute intervals between the blocks. However, healthy animals tend to swing approximately 50% to either side, animals with a unilateral cerebral lesion usually present with a dominant/biased swing direction to the contralateral side. 24 The percentage of right-side swings in 20 trials was quantified.

### Statistical Analysis

Statistical analysis was performed using OriginPro 2018 software. The data were expressed as mean ± standard error of the mean. When more than one group was compared with one control, the significance was evaluated using one-way analysis of variance with Bonferroni's post-hoc test. *p* < 0.05 was considered statistically significant.

## Results

### Anticoagulant, Antithrombotic Activities, and Bleeding Tendency Caused by the Oral Anticoagulants

**Fig. 2:**
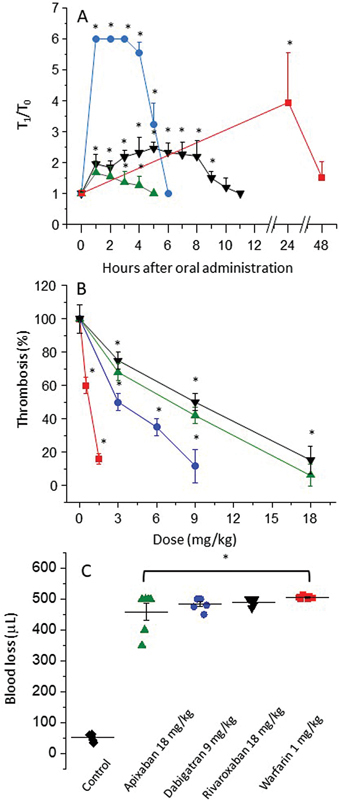
Profiles of the anticoagulant and antithrombotic activities and bleeding tendency after oral administration of anticoagulants to rats. ( **A** ) Rat plasmas were collected at different times after oral administration of 9 mg/kg dabigatran etexilate (blue); 18 mg/kg apixaban (green); 18 mg/kg rivaroxaban (black); and 1 mg/kg warfarin (red). Anticoagulant activity based on TT (blue line) or PT (red, black, and green lines) was expressed as *T*_1_ / *T*_0_ , which is the ratio of clotting time in the presence or absence of drug ( *n* = 5, mean ± SEM, * *p* < 0.05 versus control). ( **B** ) Dose dependence of the antithrombotic effect of warfarin and NOACs on the venous thrombosis model using thromboplastin as thrombogenic stimulus. ( **C** ) Assessment of bleeding tendency caused by oral anticoagulants based on the amount of blood loss after a tail injury ( *n* = 6, mean ± SEM, * *p* < 0.05 vs. control). The same doses of the anticoagulants were used in the assays of panels (A) and (C). NOACs, new oral anticoagulants; PT, prothrombin time; SEM, standard error of the mean; TT, thrombin time.

Fig. 2A shows the time course of ex vivo anticoagulant activity of the different NOACs and warfarin after oral administration. The maximum anticoagulant effect of the NOACs is achieved in approximately 1 hour. In contrast, warfarin requires approximately 24 hours to achieve its maximum anticoagulant effect in rats, as well established in the literature. 25 The numerical differences observed on the ordinate axis of Fig. 2A reflect variations in the sensitivity of TT and PT, which are the assays used to follow inhibitors of thrombin (dabigatran etexilate) and factor Xa (apixaban and rivaroxaban) or vitamin K antagonist (warfarin), respectively. 26

Subsequently we performed dose–response assays using a venous thrombosis model ( Fig. 2B ). Different doses of the anticoagulants were administered orally and the assays were performed at the time required to achieve the maximum effect ( Fig. 2A ). Both direct factor Xa inhibitors, apixaban (green), and rivaroxaban (black) have a similar antithrombotic profile, consistent with the fact that animals treated with a dose of 18 mg/kg of either of these drugs showed about approximately 90% reduction in thrombus formation. On the other hand, dabigatran etexilate (blue) and warfarin (red) showed a reduction of nearly 90% in thrombus weight at doses of 9 and 1.5 mg/kg, respectively.

Finally, we tested the bleeding tendency caused by the anticoagulant drugs at the dose and time required to achieve their maximum effects. Blood loss was extensive, about approximately 10-fold greater with the four anticoagulants than in the control animals, treated with saline ( Fig. 2C ).

### Analysis of Intracranial Bleeding Expansion Caused by Oral Anticoagulants: Ex Vivo MRI and H&E Staining

The bleeding tendency reported in the assays of Fig. 2C is not appropriate for evaluating organ-specific bleeding. Drugs may have different effects on the gastrointestinal tract or in intracranial bleeding, as reported. 27 To overcome this ambiguity, an assay to assess expansion of intracranial bleeding was employed using a specific experimental model based on collagenase-induced hemorrhage. The doses and the time to achieve the maximum effect of each drug were matched according to the assays of Fig. 2(A , B) and are shown in Table 1 .

**Table 1:** Dose and time required to reach the maximum anticoagulant and antithrombotic effects of oral anticoagulants

Drug	*T*_max_ (h)	Dose (mg/kg)	%Thrombosis
Warfarin	∼24 a	1.5	6.11 ± 6.08
Dabigatran etexilate	∼1	9	15.7 ± 3.23
Rivaroxaban	∼1	18	19.2 ± 8.22
Apixaban	∼1	18	11.7 ± 11.1

a As described in the literature. 25

**Fig. 3:**
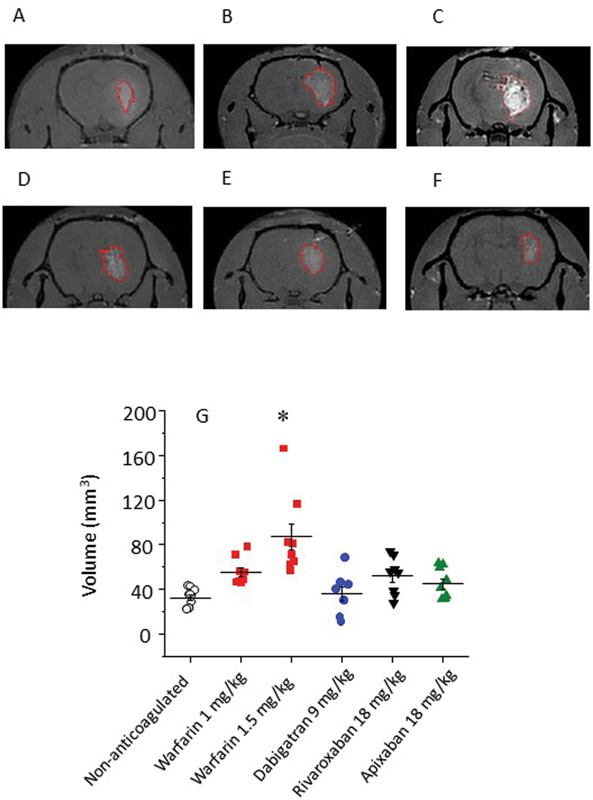
T1-weighted sequences obtained in a 2-Tesla MRI scanner 24 hours after ICH induced by collagenase injection. Representative images obtained for nontreated animals ( **A** ), warfarin at the dose of 1.0 ( **B** ) and 1.5 mg/kg ( **C** ), 9 mg/kg dabigatran etexilate ( **D** ), 18 mg/kg rivaroxaban ( **E** ) or apixaban ( **F** ) are shown in the panels. Red circles show the contour of the hematoma made using MIPAV software. Panel ( **G** ) shows the quantification of hematoma volume based on T1-weighted ( *n* = 8, mean ± SEM, * *p* < 0.05 vs. nonanticoagulated group). ICH, intracranial hemorrhage; MRI, magnetic resonance imaging.

The hematomas were initially analyzed by ex vivo MRI. Fig. 3 shows representative images of hemorrhagic lesions obtained by T1-weighted MRI. Quantification of lesion volumes using MRI images ( Fig. 3B ) shows that warfarin increases intracranial bleeding compared with nontreated animals, especially at the dose of 1.5 mg/kg (87.11 ± 34 vs. 32.02 ± 8.27 mm ^3^ , respectively). On the other hand, NOACs do not lead to a significant increase in intracerebral bleeding, as revealed by ex vivo MRI analysis. Animals treated with dabigatran etexilate at the dose of 9 mg/kg, and apixaban and rivaroxaban at 18 mg/kg present average bleeding volumes of 35.92 ± 18.4, 44.90 ± 13.8, and 51.68 ± 16.8 mm ^3^ , respectively, similar to controls.

**Fig. 4:**
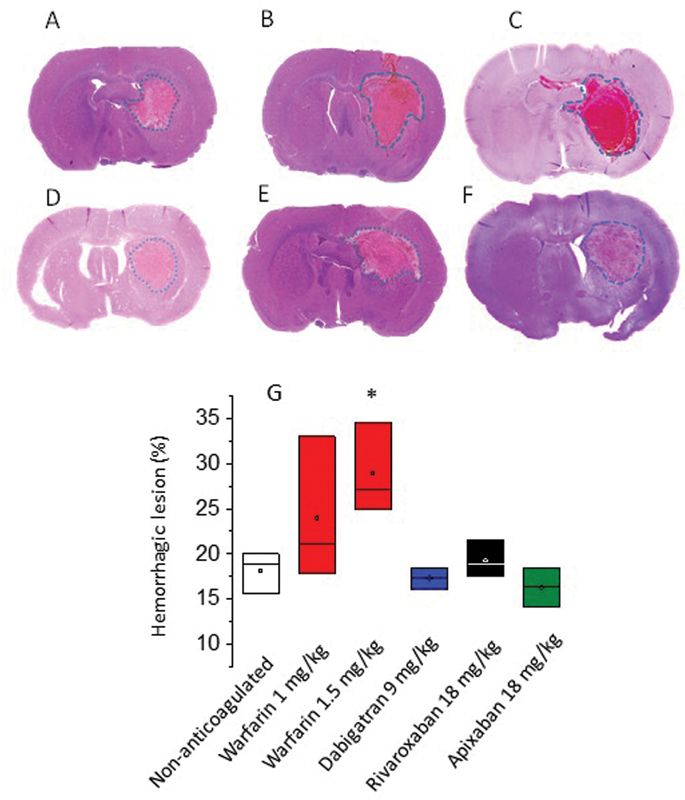
Images of the histological analysis of the hemorrhagic areas observed on nontreated ( **A** ) or anticoagulated ( **B–F** ) animals using H&E staining. Quantitative data are shown in panel ( **G** ). The anticoagulant doses were 1 mg/kg warfarin (B), 1.5 mg/kg warfarin (C), 9 mg/kg dabigatran etexilate (D), 18 mg/kg rivaroxaban (E), or apixaban (F). (G) Quantification of the hemorrhagic lesion (%) in relation to the whole ipsilateral hemisphere ( *n* = 3, mean ± SEM, * *p* < 0.05 vs. nonanticoagulated group). SEM, standard error of the mean.

These results were complemented by histological analysis, which confirmed that the control group presented an average of 18.13 ± 2.7% of hemorrhagic lesion in relation to the area of the ipsilateral hemisphere, while the groups treated with warfarin presented areas of 23.97 ± 2.9 and 28.86 ± 3.9% at doses of 1 and 1.5 mg/kg, respectively. Regarding the NOACs, we observed an average of 17.66 ± 2.1, 19.12 ± 2.04, and 16.27 ± 2.1% in animals treated with dabigatran etexilate, apixaban, and rivaroxaban, respectively ( Fig. 4G ).

### Evaluation of the Blood–Brain Barrier Breakdown by Evans' Blue Extravasation

**Fig. 5:**
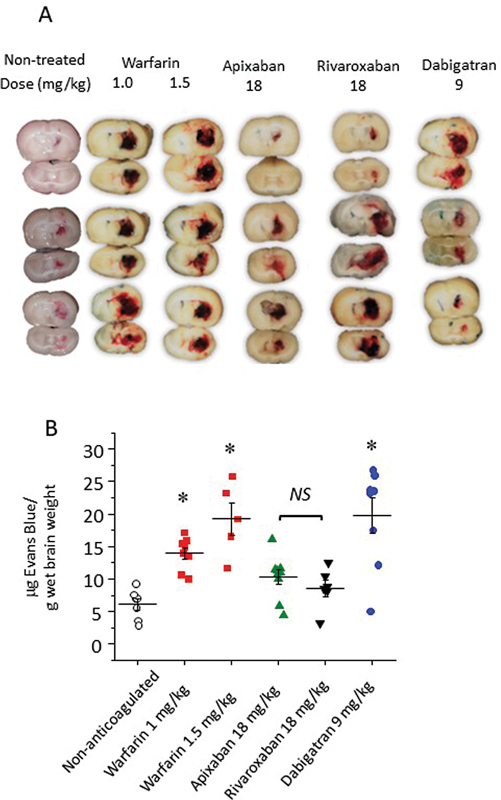
Evans blue extravasation from the brains of rats treated with saline or oral anticoagulants after 24 hours of collagenase-induced ICH. Evans blue was injected intravascularly and allowed to circulate for 30 minutes. After perfusion, animal was sacrificed and the amount of Evans blue extravasated into the brain was quantified by spectrophotometric analysis. Panel ( **A** ) shows representative macroscopic images with the left hemisphere uppermost in each pair, while quantitative data are in panel ( **B** ) ( *n* = 8, mean ± SEM, * *p* < 0.05 vs. nonanticoagulated group). ICH, intracranial hemorrhage; SEM, standard error of the mean.

We complemented MRI and histological analysis of intracranial bleeding using an additional assay based on Evans blue extravasation. Representative images of the dye extravasation in the brain of animals treated with different anticoagulants are shown in Fig. 5A , while quantitative data appear in Fig. 5B . Treatment with warfarin at doses of 1 and 1.5 mg/kg significantly increased Evans blue extravasation compared with control, nontreated animals (13.9 ± 2.5 and 19.28 ± 5.5 µg/g vs. 6.11 ± 2.2 µg/g, respectively; Fig. 5B ). Surprisingly, there was a significant increase in dye extravasation in animals treated with dabigatran etexilate at 9 mg/kg (19.73 ± 7.6 µg/g), but not with apixaban or rivaroxaban (10.29 ± 3.4 and 8.57 ± 3.1 µg/g, respectively).

### Neurological Evaluation

**Fig. 6:**
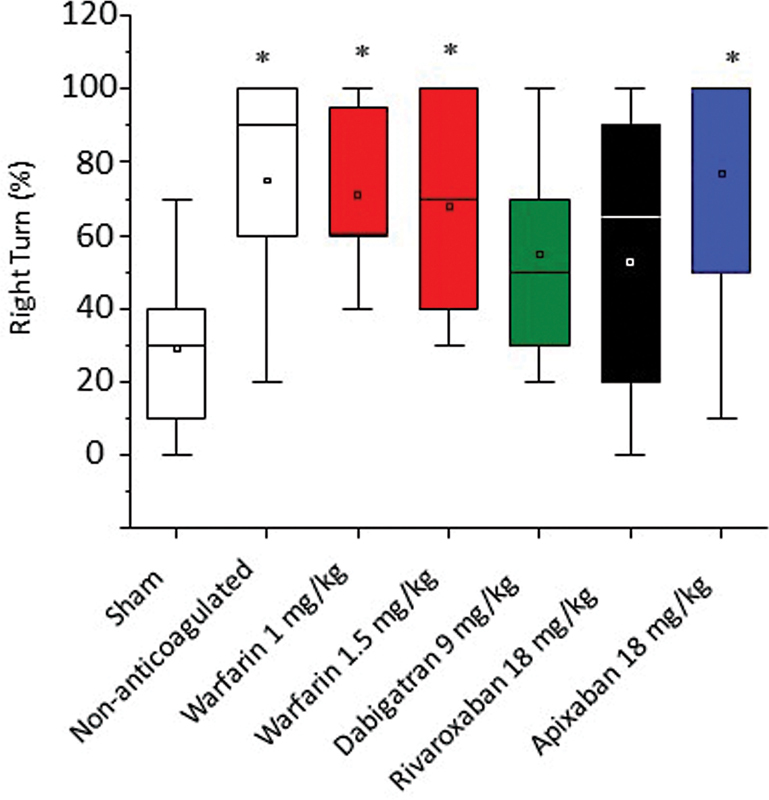
Assessment of motor function by the elevated body swing test. Percentage of right turns in the elevated body swing test was assessed 24 hours after induction of the ICH model ( *n* = 8, mean ± SEM, * *p* < 0.05 vs. sham group). ICH, intracranial hemorrhage; SEM, standard error of the mean.

The elevated body swing test revealed that, compared with the sham group, animals with ICH had an increased percentage of right-side swings 24 hours after collagenase injection. However, no significant differences were found among the four anticoagulant groups ( Fig. 6 ). Warfarin-anticoagulated animals subjected to the ICH were very debilitated, with difficult locomotion, bleeding from the eyes and nostrils, and several of them died (35%). Animals anticoagulated with NOACs occasionally showed hemorrhages in feces and urine, but had mortality rates like controls, nontreated animals (10%).

## Discussion

ICH is the most dangerous complication associated with anticoagulant drugs, usually with high mortality and poor prognosis. 11 Due to the variable dose–response and high incidence of ICH associated with the use of warfarin, NOACs were developed and entered the world market during the last two decades. 2 However, data on the increased risk of ICH associated with the use of NOACs are still controversial. 6 7 8 9 10 11 12 13 In this work, we explore this topic using a translational approach based on a model of ICH induced by collagenase injection into the striatum of rats previously treated with different oral anticoagulants.

After determining the time to reach maximum anticoagulant and antithrombotic activities ( Figs. 2A, B ), we used an experimental model of ICH in rats based on collagenase injection into the left striatum. Quantification of bleeding volume using T1-weighted images obtained by ex vivo MRI showed that warfarin significantly increases bleeding compared with control, nonanticoagulated animals, especially at 1.5 mg/kg ( Fig. 3 ). We did not observe an increase of intracerebral bleeding in animals treated with NOACs in our protocol. These data corroborate histological analysis ( Fig. 4 ), since the same animals were used in both assays.

Regarding the breakdown of the blood–brain barrier, Evans blue extravasation has been the assay most used by different authors to correlate with the volume of bleeding due to the strong binding of this dye to plasma albumin. 28 Quantification of the Evans blue extravasation showed a significant increase in both warfarin- and dabigatran etexilate-treated animals ( Fig. 5 ). Thrombin plays an important role in cerebrovascular events, with both protective and neurotoxic actions. 29 After a hemorrhagic insult, the conversion of fibrinogen to fibrin and its procoagulant action are fundamental in limiting the expansion of intracerebral bleeding. More studies are needed to ascertain whether the selective inhibition of thrombin by dabigatran etexilate could compromise the permeability of the blood–brain barrier. In addition, the Evans blue method could detect changes in the permeability of the blood–brain barrier that are not detected by MRI.

Data from the literature about the effects of NOACs on ICH using animal models have shown contradictory results. Probably the controversies derive from the methods used to induce or quantify ICH in addition to the dose and route for administration of NOACs. Lauer et al 30 reported that orally administered anticoagulation with dabigatran at very high doses (up to 112 mg/kg) did not increase ICH in CD-1 mice. In the study by Lauer et al, the dose of collagenase used to induce cerebral damage was 0.2 units and the analysis of bleeding was based exclusively on quantification of hemoglobin by a spectrophotometric method. In another case, oral doses of 10 and 20 mg/kg of dabigatran also did not increase ICH using the same 0.2 unit dose of collagenase, but bleeding was quantified solely by MRI. 31 This last study corroborates our findings about the effect of NOACs on ICH using MRI. However, increased ICH was detected by MRI when dabigatran etexilate was administered intraperitoneally at doses of 2.25 to 9 mg/kg. 32 An increase of ICH was observed when rivaroxaban was orally administered at 30 mg/kg and when high doses of collagenase were used. No effect was observed with only 10 mg/kg of the same NOAC. 33 Low doses of collagenase cause less intense and more resolvable bleeding, which is likely to influence the effect of NOACs expanding ICH in an animal model.

The volume of hematoma is a strong predictive factor for morbidity and mortality. 34 Prevention of early hematoma growth is an important therapeutic strategy for acute ICH and current approaches are restricted to reduction of the blood pressure and reversal of the anticoagulant effect using different strategies. 35 Specific antidotes for NOACs have become available in recent years but more robust clinical studies are required to ensure the efficacy of these new drugs in limiting expansion of ICH and to improve the prognosis of the patients. 36 The bleeding site also influences the prognosis, 37 and it is an important aspect to consider in standardization of appropriate methods to evaluate hematomas' volume and their expansion.

The evaluation of ICH should not be restricted to morphological analysis of the extension of the hematoma. These data can be complemented by assessing the impact of ICH on neurological symptoms and response to neuromotor and behavioral tests. 38 This approach helps to evaluate the impact of different anticoagulants on ICH and its consequences. With this in mind, we tested neuromotor alterations using the elevated body swing test. There was an increased tendency to rotate to the contralateral side in the injured rats but no statistical difference among the anticoagulated animals ( Fig. 6 ). In a microhemorrhage model in mice, changes in long-term cognitive activities, such as a reduction in visual recognition memory, were observed, 39 but not in spontaneous motor activity, while another work has shown that neuromotor changes can occur 3 days after the hemorrhagic insult. 40

Our work has limitations. We used doses of NOACs much higher than those used in clinical practice. Healthy rats were used in the experiments, and high blood pressure is the main risk factor for ICH. 41 Future experiments using different doses of NOACs in healthy and hypertensive animals are needed to evaluate the expansion of ICH associated with anticoagulant therapy. In the elevated body swing test, animals treated with dabigatran and rivaroxaban did not show statistical differences in relation to the sham group. This indicates that further studies using different neuromotor and behavioral assessments at several periods of time after brain injury will help to understand the impact of anticoagulants on the possible sequelae of ICH.

In recent years, several studies have focused on the mechanism of secondary inflammation, which can cause cerebral edema. 42 Neuroinflammation has recently been associated with poor functional outcomes in patients afflicted with a multitude of neurological conditions, including ICH. In addition to its anticoagulant property, heparin has significant neuroprotective and anti-inflammatory effects, especially in subarachnoid hemorrhage. 43 44 It seems contradictory to propose the use of a drug with potent anticoagulant action as a therapeutic alternative for ICH. However, derivatives of heparin obtained by chemical modifications or even from natural sources have been proposed as alternatives for use in events not related to blood coagulation. 45 46 The additional beneficial effects of heparin are preserved in these derivatives, which are devoid of anticoagulant action. A low-anticoagulant heparin obtained from bovine intestine may be an interesting candidate for additional tests. 47

In conclusion, we compared the effects of different NOACs and warfarin on the expansion of ICH using an experimental model based on collagenase injection in the brain striatum, leading to rupture of blood vessels and bleeding into adjacent tissues. This model reproduces experimentally the effects of spontaneous ICH in humans. The expansion of the hematomas was evaluated by MRI, Evans blue extravasation, and histological examination. Our results indicate that NOACs do not increase intracranial bleeding compared with nontreated animals, while warfarin markedly favors the expansion of the hematomas. Only dabigatran etexilate caused a modest but significant increase in the assay of Evans blue extravasation. Translational approaches combining different standardized methods to induce ICH and to evaluate the consequences after administration of NOACs will help the comprehension of the clinical phenomena that accompany ICH, result in high mortality rates, and are still poorly elucidated.

## Notes

**What Is Known about This Topic?**

- Intracerebral hemorrhage (ICH) is the most serious complication of anticoagulant therapy.
- The effect of different anticoagulant drugs on hematoma expansion in patients with ICH remains a topic of intense debate, especially for the new oral anticoagulants (NOACs).
- Experimental models are alternatives to evaluate the impact of these drugs on ICH.

**What Does This Paper Add?**

- The effects of NOACs on the expansion of ICH were evaluated using an experimental model of collagenase-induced ICH in rats. Warfarin was also tested for comparison. Expansion of the hematomas was evaluated by a variety of methods, including ex vivo magnetic resonance imaging, histological analysis, Evans blue extravasation, and neuromotor test.
- NOACs did not increase ICH compared with nontreated animals while warfarin markedly expanded the hematomas in MRI and histological analysis. In contrast to factor Xa inhibitors, dabigatran etexilate and warfarin increased Evans blue leakage.
- Our translational approach indicates a favorable effect of NOACs on the expansion of ICH compared with warfarin.
